# Editorial: Understanding stroke recovery to improve outcomes: From acute care to chronic rehabilitation

**DOI:** 10.3389/fneur.2022.1021033

**Published:** 2022-09-27

**Authors:** Adriana Bastos Conforto, Sook-Lei Liew, Andreas R. Luft, Tomoko Kitago, Julie Bernhardt, Juan Francisco Arenillas

**Affiliations:** ^1^Hospital Israelita Albert Einstein, São Paulo, Brazil; ^2^Hospital das Clínicas/São Paulo University, São Paulo, Brazil; ^3^University of Southern California, Los Angeles, CA, United States; ^4^Center for Neurology and Rehabilitation (CERENEO), Vitznau, Switzerland; ^5^University Hospital Zürich, Zurich, Switzerland; ^6^Burke Neurological Institute, White Plains, NY, United States; ^7^Weill Cornell Medicine, New York, NY, United States; ^8^Florey Institute of Neuroscience and Mental Health, University of Melbourne, Parkville, VIC, Australia; ^9^Hospital Clínico Universitario de Valladolid, Valladolid, Spain

**Keywords:** recovery, plasticity, stroke, multidisciplinary, neurorepair

Over the past decades, stroke outcomes have improved due to advances in treatment including the implementation of reperfusion therapies such as thrombolysis and mechanical thrombectomy for acute stroke and multidisciplinary care in stroke units (1, 2). In addition, progress in research about brain function, repair mechanisms and rehabilitation interventions informed by both animal and human studies, is helping shape both research and practice (3). Yet, stroke remains a major cause of death and disability worldwide (4). The integration of data about the predictors of recovery and responsiveness to interventions, as well as the impact of psychosocial factors such as motivation and self-efficacy, has the potential to decrease the burden from this condition.

The Research Topic “Understanding Stroke Recovery to Improve Outcomes: From Acute Care to Chronic Rehabilitation” included 30 manuscripts, consisting mainly of observational studies but also of proof-of-principle randomized trials, two narrative reviews, a systematic review, a meta-analysis, a study protocol and a case report. Most of the studies addressed the prediction of outcomes or effects of specific interventions on behavioral, neurophysiological and imaging metrics.

The prediction of risks of complications in the acute phase, as well as the prognostication of long-term outcomes can influence goals of care, selection of treatment strategies as well as expectations of patients and their families. Lin et al. proposed models to predict deterioration during hospitalization and prognosis at 1-year after intracerebral hemorrhages, to be validated by prospective studies in the future. Wurzinger et al. analyzed longitudinal data from a research database and showed that Barthel Index scores within the first 2 days after stroke predicted self-reported dependency in performance of activities of daily living, at 3 months as well as 12 months after stroke.

Shen et al. investigated risk factors and prognosis of symptomatic intracranial hemorrhage (sICH) in patients admitted within 1 week after ischemic stroke, not treated with intravenous thrombolysis or thrombectomy, and included in the Chinese Acute Ischemic Stroke Treatment Outcome Registry (CASTOR), a large multicenter, prospective study. Modified Rankin Scale (MRS) scores were assessed at 3 and 12 months after onset of symptoms. sICH occurred in 0.73% of the patients. This rate is similar to that reported in patients not treated with alteplase in the NINDS trial (0.6%) (5). Three variables were independently associated with sICH: atrial fibrillation, history of tumors, and NIHSS score at admission. SICH was associated with higher risk of poor outcome at 3 months, but not at 12 months, and with increased mortality at 3 and 12 months.

Upper limb paresis is frequent and can substantially impact disability after stroke. Pradines et al. showed in 80 stroke survivors, at a median of 9 years after stroke-onset, that chronic muscular shortening is greater in lower than upper limbs, but weakness is more prominent in the arms. In a review article, Ballester et al. condensed the evidence for a link between arm use and arm recovery: if the arm is used in daily life above a certain threshold, it is recovering through self-training creating a virtuous circle between use and recovery. If not, the opposite is triggered resulting in a vicious circle.

Three studies aimed to predict upper limb motor impairments or disability. Ueda et al. followed 60 patients and concluded that manual muscle testing of elbow flexion and active finger extension up to 72 h after stroke may be useful to predict Fugl-Meyer Assessment upper extremity motor scores and Action Research Arm Test scores, at 3 weeks. da Silva et al. combined clinical testing at admission to acute inpatient rehabilitation and MRI metrics to predict upper limb performance at a later stage after stroke. They estimated shoulder abduction and finger extension (E-SAFE) scores according to medical records, and extracted metrics of corticospinal tract (CST) lesion from routine, standard of care brain MRI, in 34 patients who completed acute inpatient rehabilitation post-stroke. CST lesion overlap was depicted by means of spatial normalization of lesion masks that were then overlaid onto a white matter tract atlas delineating CST contributions from six cortical seed regions. The authors found that upper limb performance at a median time of 3 months may be predicted by the combination of E-SAFE scores performed at a median time of 7 days, and the percentage of CST lesion overlap on MRI performed at a median of 1 day post-stroke. Interestingly, MRI metrics of CST lesion, especially those involving projections from the ventral and dorsal premotor cortices, were able to classify upper limb outcomes at a median time of 3 months, with 79.4% accuracy. The combination of clinical and MRI variables increased accuracy to 88.2%.

Neurological impairments other than upper limb paresis, but also relevant to disability, were targeted by four manuscripts. Verbeek et al. performed external validation of the Early Prediction of Functional Outcome after Stroke (EPOS) model for independent gait at 3 months post-stroke. This model was originally designed to evaluate patients between days 2 and 9, in order to predict gait independence at 6 months post-stroke. EPOS performances on days 3, 8, and 9, but not on day 1, were acceptable to predict independent gait at 3 months in mild to moderately affected patients with first-ever stroke and no pre-stroke disability. Possible next steps include the use of this model in clinical practice, in patients with a similar profile, and to test its predictive value in subjects with more severe or recurrent strokes.

Serrada et al. followed 89 patients with motor impairments up to 6 months after stroke and observed that recovery of upper limb sensation and body awareness predominantly occurred within the 1st month. In addition, sensation and body awareness were correlated not only with motor impairment and quality of life but also with self-efficacy, the belief in the capacity to achieve certain outcomes. In another manuscript, Gangwani et al. reviewed the underpinnings and the role of self-efficacy in stroke recovery, summarizing the potential for further research and development of novel interventions to improve outcomes.

Lucente et al. followed 359 subjects after acute stroke and found that fecal incontinence was present in 2% of the first-ever anterior circulation strokes. Hemorrhagic stroke and higher NIHSS scores were independently associated with the presence of fecal incontinence in the acute phase. The condition persisted in 44% of the subjects, at 3 months.

Together, these studies indicate that assessments performed early after stroke can be useful to forecast outcomes related to body structure and function, activity or participation. Not only prognostication of recovery, but also of responsiveness to rehabilitation strategies are relevant in clinical practice, so that personalized therapeutic plans can be designed. In a retrospective study, Goffredo et al. reported that a subset of kinematic parameters predicted motor impairment after upper-limb Robot-assisted Therapy in 66 subjects in the subacute phase after stroke. These parameters may hence be useful to identify patients more likely to benefit from this type of intervention.

One of the goals of rehabilitation is to positively influence recovery trajectories. Otero-Ortega et al. outlined the state of the art on the use of trophic factors, cell therapy, and extracellular vesicles to promote adaptive plasticity. Hung et al. discussed the exciting perspective of protecting the brain prior to a lesion. They proposed that pre-stroke physical activity could enhance collateral circulation, known to play a crucial role in protecting the brain against infarction during ischemia. According to this hypothesis, physical activity may limit the extent of infarction and increase the odds of good outcomes after first-ever or recurrent ischemic strokes.

Other promising strategies to boost recovery were covered by several manuscripts. The interventions administered to achieve this goal included administration of sertraline, intensive rehabilitation, cycling combined with functional electric stimulation, acupuncture and neuromodulation interventions. Disability, upper or lower limb motor impairment/function, dysphagia, cognitive and visual outcomes were evaluated.

Pharmacological enhancement of recovery motivated several investigations over the past decades. In a prospective observational study of consecutive acute ischemic stroke patients and motor impairments (*n* = 114), Stuckart et al. reported a higher rate of favorable outcomes (mRS **≤** 2 at 3 months) in those who received sertraline at the discretion of the treating neurologist—for instance, for clinically suspected post-stroke depression or at high risk for this condition—compared to an untreated control group.

Intensity, frequency and starting time of rehabilitation may be critical to stroke outcomes. Garcia-Rodrigues et al. followed three cohorts for 6 months: patients with stroke treated with (1) intensive rehabilitation therapy or (2) conventional therapy, and control subjects without stroke. Upper limb motor impairments and functional ambulation improved earlier in patients treated with intensive rehabilitation therapy.

The definition of doses or therapeutic windows for optimal delivery of rehabilitation interventions, as successfully performed in the field of hyperacute stroke treatment (1, 2) may harness the development of game-changing interventions and inform clinical trials. Kroth et al. described the protocol of a study that intends to compare effects of repetitive peripheral sensory stimulation delivered in combination with upper limb training, delivered either at an early phase or in the chronic phase after stroke, on upper limb motor outcomes and imaging biomarkers.

In a study by Hu et al., training in the form of cycling was combined with functional electrical stimulation, in a non-controlled sample of 15 stroke survivors. The authors observed improved electromyographic recruitment that correlated with improved scores on balance and ambulation, after treatment. A different training paradigm was employed by Awosika et al. in order to improve gait. They reported that backward locomotor treadmill training (BLTT), consisting of training on an instrumented treadmill without body-weight support (*n* = 39), led to improvements in measures of spatial walking.

In a meta-analysis of randomized controlled studies comparing standard treatment plus and minus scalp acupuncture, Huang et al. found a benefit of additional acupuncture on motor function. Three months of acupuncture had a greater effect than 1 month. Which aspect of acupuncture (sensory stimulation, psychological factors) caused the effect, remains to be investigated.

Balcerak et al. systematically reviewed 41 randomized controlled trials to address dysphagia in subacute stroke. Interventions included acupuncture, physical therapy, drug therapy, neuromuscular electrical stimulation, pharyngeal electrical stimulation, transcranial direct current stimulation and repetitive transcranial magnetic stimulation (rTMS). Of these, intensive physical therapy, rTMS and pharmaceutical treatment are promising; however, further research is required.

Neuromodulation interventions are promising therapeutic treatment for addressing multiple domains of recovery after stroke. Kim et al. showed that high-frequency rTMS over the ipsilesional dorsolateral pre-frontal cortex improved Mini-Mental status exam, Functional Independence Measure-cognition subscale, and forward Digit Span compared to a sham control group in subacute stroke patients. These results varied based on the lesioned hemisphere.

The application of inhibitory rTMS to contralesional Broca's area has emerged as a promising intervention to promote language recovery after stroke. Lin et al. investigated the efficacy of inhibitory rTMS over the contralesional pars triangularis, and associated functional connectivity changes in patients with chronic poststroke non-fluent aphasia, in a randomized controlled trial. Following 10 daily sessions of rTMS, they found significant improvement in language performance in the rTMS group compared with the sham stimulation group. Using resting-state fMRI, they also demonstrated changes in functional connectivity, including increased connectivity in perilesional and spared language areas of the left hemisphere, and reduced connectivity in right hemisphere language relevant areas, in particular the right pars triangularis and pars opercularis. Interestingly, they found that specific functional connectivity changes could predict language improvements following rTMS. This study provides important insights into the mechanisms underlying language improvement with contralesional rTMS over Broca's area, supporting the theory of interhemispheric imbalance during post-stroke recovery of aphasia.

On the other hand, the role of the contralesional primary motor cortex (M1) after stroke is still highly debated. Dionisio et al. showed that continous theta burst stimulation over the contralesional motor cortex resulted in an excitatory effect in the contralesional motor cortex after stimulation, but did not report significant changes to motor behavior. Revill et al. examined whether increased contralesional M1 activation, which is consistently observed in imaging studies after stroke, is due to increasing demand due to impaired motor ability. In this fMRI study, they varied the precision requirements in a hand motor task and demonstrated that with increasing task demand, there was stronger activation of the contralesional M1 in both stroke patients and healthy age-matched controls, though patients were less likely to show a linear relationship in the contralesional M1 with increased task difficulty compared with controls. These findings highlight the importance of considering task demand in studies aimed at understanding the role of the contralesional M1 and in the development of interventions that target this area for neurorehabilitation.

Connectivity studies can provide clues about mechanisms of reorganization as well as effects of interventions. Xu et al. investigated effects of neuromodulation in 24 patients with unilateral occipital strokes and hemianopia. Using resting-state electroencephalogram, they reported increased functional connectivity between the occipital and temporal lobes in the contralesional hemisphere after delivering cathodal transcranial direct current stimulation of the contralesional hemisphere, combined with transcranial alternating current stimulation. Huang et al. performed fMRI-based connectivity analysis and described disruptions in global connectivity after stroke as compared to the healthy brain. Connectivity in certain brain areas (occipital cortex) in right-sided stroke survivors correlated with arm impairment and its recovery (as measured using the Fugl-Meyer Assessment).

On the other hand, Boot et al. found no associations between post-stroke fatigue, known to be associated with worse functional outcomes, and imaging metrics (lesion size, site or metrics of brain network connectivity) in young patients with stroke.

Bridging the gaps between knowledge gained about mechanisms of plasticity, prediction of outcomes, relationships between behavior and neurophysiological or imaging biomarkers, and development of interventions that lead to meaningful effects from the perspective of persons affected by stroke, is a major challenge for rehabilitation science. This Research Topic combined efforts from investigators engaged across the stroke continuum of care.

Factors and pathways determining the probability of recovery from an acute stroke are acting from the same moment of stroke onset and even before (prior conditioning by exercise), so knowledge of these factors by acute stroke teams responsible for patients care during the acute phase may be essential to improve outcomes. The concept that stroke recovery should be taken care of only after hospital discharge is clearly challenged by the evidence provided in this article collection.

Further research is needed to better characterize and design the process of care combining multidisciplinary acute phase and post-acute phase interventions aimed to optimize recovery from stroke. We have a well-designed hyperacute process of care (stroke code system), with coordinated multidisciplinary effort, and probably a similar innovating effort in post-stroke process of care may be needed to obtain the best effect from the neurorepair therapeutic strategies presented in this collection.

There may be important challenges ahead to increase the level of evidence for these interventions in order to be transferred into clinical practice. One important step might be to increase awareness across the stroke continuum of care as a whole, having long-term functional outcome and quality of life as the main drivers of value for all actors in the process: patients, healthcare professionals, institutions, and ultimately society.

## Author contributions

AC, S-LL, TK, AL, and JA wrote sections of the manuscript. AC wrote the first draft of the manuscript. All authors contributed to manuscript revision, read, and approved the submitted version.

## Conflict of interest

The authors declare that the research was conducted in the absence of any commercial or financial relationships that could be construed as a potential conflict of interest.

## Publisher's note

All claims expressed in this article are solely those of the authors and do not necessarily represent those of their affiliated organizations, or those of the publisher, the editors and the reviewers. Any product that may be evaluated in this article, or claim that may be made by its manufacturer, is not guaranteed or endorsed by the publisher.
